# Seroprevalence of Toxoplasma gondii Antibodies and Associated Risk Factors Among Women in Zakho City, Iraq

**DOI:** 10.7759/cureus.56328

**Published:** 2024-03-17

**Authors:** Kalthom M Mustafa, Ahmed B Mohammed, Wijdan M.S. Mero

**Affiliations:** 1 Department of Biology, Faculty of Science, University of Zakho, Zakho, IRQ; 2 College of Science, Nawroz University, Duhok, IRQ

**Keywords:** risk factors, females, toxoplasmosis, igg and igm antibodies, seroprevalence

## Abstract

Toxoplasmosis, an infectious disease caused by the obligate intracellular protozoan parasite, poses varying degrees of risk, ranging from asymptomatic cases in immunocompetent individuals to severe, life-threatening conditions in immunocompromised individuals and developing fetuses, especially when infection occurs during early pregnancy. While the disease is endemic in Iraq, there is a notable lack of precise information regarding its seroprevalence among females of childbearing age and pregnant women, along with associated risk factors in the Zakho district.

This cross-sectional study aimed to address this gap by determining the prevalence of anti-*Toxoplasma gondii* IgG and IgM antibodies using the ELISA assay. The study involved 610 females aged 18-79 years from various residential areas within Zakho district, Iraq. The findings revealed a seroprevalence of 32.46% for anti-*Toxoplasma* IgG antibodies and 8.86% for IgM antibodies.

Significant variations in IgG antibody seroprevalence were observed across different age groups (P=0.008), with the highest prevalence noted among those aged 46-55 years (47.73%). Conversely, IgM antibody seroprevalence, while non-significant (P>0.05), displayed the highest rate of 10.05% among ages 18-25 years.

The study identified residence as a variable significantly associated with toxoplasmosis. Additionally, contact with cats, marital status, a history of abortion, and the consumption of homemade food showed significant associations with anti-*Toxoplasma *IgM antibodies only.

These findings strongly suggest that *Toxoplasma gondii *is a prevalent causative agent of infection in Zakho city, Iraq. This study contributes valuable insights into the seroprevalence and associated risk factors, providing a foundation for targeted interventions and further research in this region.

## Introduction

Toxoplasmosis is an infectious disease caused by the intracellular protozoan parasite *Toxoplasma gondii *(*T. gondii*). This infection is clinically significant in immunocompromised individuals and pregnant women at the early pregnancy stage [1,2]. Human infection primarily occurs through two significant routes: oral transmission and transplacental transmission [3]. Typically, this parasite is transmitted to humans through consuming food or water contaminated with sporulated oocysts or through the consumption of undercooked meat containing tissue cysts. Furthermore, other rare methods that lead to acquiring toxoplasmosis include the introduction of infected blood or leukocytes and organ transplantation [4,5].

Toxoplasmosis during pregnancy can lead to profound consequences, particularly affecting both the mother and the developing fetus, resulting in congenital toxoplasmosis [6]. The complications linked to this parasitic infection encompass a spectrum of adverse outcomes, such as miscarriage, severe abnormalities, developmental delays, hydrocephalus, intracerebral calcification, blindness, epilepsy, and, in certain cases, intrauterine fetal death (IUFD) [7].

Due to its asymptomatic nature, limited resources and comparatively lower priority are directed to this infection in healthcare policies, as it is commonly excluded from regular screening or surveillance programs [8]. Therefore, quantifying the prevalence of congenital toxoplasmosis poses a greater challenge compared to assessing the seroprevalence of *T. gondii*. The prevalence of infection in humans has been documented variably globally. Studies performed in Iraq exhibited significant diversity in the seroprevalence of anti-*T. gondii* IgG antibodies among women. The seroprevalence rates are as follows: in Zakho 12.21%, in Duhok 17.6%, in Basra 30.8%, in Baghdad 31.5% [9-12], in Turkey 23.2% [13], in Iran 38.8% [14], in Saudi Arabia 8.3% [15], and in Pakistan 25.8% [16]. 

Serological and molecular investigations have been conducted globally, revealing that over 33% of the population carries antibodies against *T. gondii* [17-19]. Considering the uncertainty in our knowledge regarding the exact seroprevalence of toxoplasmosis among females in Zakho and the substantial variations in the reported results across different areas, it is imperative to commence a seroprevalence study specifically among females in Zakho city, Iraq, and to illustrate the presence of any correlation between the infection and some risk factors. This undertaking is crucial for acquiring precise insight into the extent of the prevalence of toxoplasmosis among women in this city in order to formulate effective preventive measures.

## Materials and methods

Study design and serological tests

This investigation was carried out as a cross-sectional study starting from May 2021 to April 2022. The study encompassed all females who sought medical care at the General Hospital of Zakho and Cham Mishko Camp Health Care in Zakho city. The sample size has been taken according to the Cohen methods [20]. A total of 610 women aged 18-79 years from various residential areas were enrolled as the study population, following the acquisition of written informed consent from each participant, in addition to the approval from the Research Ethics Committee of the College of Medicine, University of Zakho (approval number: Jun21/E01).

Data collection

Each participant completed a questionnaire (see Appendices) during sample collection, to gather information on factors such as age, occupation, location, abortion history, cat ownership, and regular consumption of restaurant foods. After obtaining consent from each participant, 5 ml of blood was aseptically withdrawn using vacutainer tubes without anticoagulants. Following collection, the samples underwent centrifugation at 3000 rpm for five minutes. The resulting serum was subsequently transferred to Eppendorf tubes. These tubes were appropriately labeled with details corresponding to the study's questionnaire. The serum samples were then transported to the biology laboratory at the University of Zakho and stored at -20°C to be used later on for ELISA assay for the estimation of anti-*Toxoplasma gondii* IgG and IgM antibodies using ELISA kit from DRG International Instruments, GmbH, Germany, according to the instruction supplied with the kit.

Data analysis

The acquired data underwent rigorous analysis using GraphPad Prism software (Version 9.0.2, 2021). A p-value less than 0.05 was set as the threshold for statistical significance, indicating robust and compelling results. To scrutinize and compare the seroprevalence values with respect to the subjects' characteristics, we employed the chi-squared tests, ensuring a thorough examination of the data.

## Results

Among the enrolled females, 32.46% (198 out of 610) tested positive for anti-*Toxoplasma* IgG antibodies in their sera, indicating exposure to *Toxoplasma gondii*. Additionally, 8.68% (53 out of 610) showed positivity for anti-*Toxoplasma* IgM antibodies, suggesting recent or acute infections (see Table 1).

**Table 1:** The seroprevalence of anti-Toxoplasma IgG and IgM antibodies among tested females in Zakho city. The provided data illustrates both the number (No.) of examined females' sera and the corresponding percentage (%) of seropositive IgG and IgM antibodies.

Gender	No. of examined samples	IgG +		IgM +	
		No.	%	No.	%
Females	610	198	32.46	53	8.68

The seroprevalence of IgG antibodies exhibited highly significant differences between distinct age groups (P=0.008). Notably, the highest prevalence was observed among individuals aged 36-45 and 46-55 years, with rates of 53 (41.73%) and 22 (47.73%), respectively. Conversely, the lowest seroprevalence, at 20 (5%), was identified among individuals aged 56-79 years. In contrast, the highest seroprevalence of IgM antibodies, at 20 (10.05%), was documented among individuals aged 18-25 years. However, statistical analysis revealed non-significant differences (P>0.05) in IgM seroprevalence across various age groups, as detailed in Table 2.

**Table 2:** The seroprevalence of anti-Toxoplasma antibodies among females in Zakho has been examined across different age groups. The data is presented as counts (No.) and percentages (%) of the total sample within each age group, including the percentages of positive IgG and IgM antibodies. Significance is attributed to a p-value less than 0.05 or 0.001, while a p-value exceeding 0.05 is considered non-significant.

Variables	Sample no.	IgG +			IgM +		
		Positive no.	%	P-value	Positive no.	%	P-value
Age group							
18-25	199	56	28.14	0.008 **	20	10.05	0.18
26-35	218	62	28.44		16	6.88	
36-45	127	53	41.73		12	9.45	
46-55	46	22	47.83		3	6.52	
56-79	20	5	25.00		2	5.00	

Table 3 illustrates the relationship between the seroprevalence of anti-*Toxoplasma* IgG and IgM antibodies and their association with various demographic variables. Significant differences were observed in the seroprevalence of anti-*T. gondii *IgG and IgM antibodies based on residence (urban, rural, and camp). Highly significant variations (P<0.04 and 0.0001) were noted in IgG seroprevalence among urban (56, 26.34%), rural (107, 36.39%), and camp residents (32, 34.78%), as well as IgM seroprevalence (12, 5.35%), (6, 2.04%), and (35, 38.04%), respectively.

**Table 3:** Seroprevalence of Toxoplasma infection among females in Zakho according to sociodemographic characteristics. The presented number (No.) in the table are the total and positive sample numbers for variables including the area of residences, contact with cats, occupation, marital status and history of abortion, and percentages (%) of the positive IgG and IgM antibodies. Significance is attributed to a p-value less than 0.05 or 0.001, while a p-value > 0.05 is considered non-significant.

Variables	Sample no.	IgG +			IgM +		
		Positive no.	%	P-value	Positive no.	%	P-value
Residence							
Urban	224	59	26.34	0.04 *	12	5.35	0.0001***
Rural	294	107	36.39		6	2.04	
Camp	92	32	34.78		35	38.04	
Contact with cats							
Yes	152	41	26.97	0.1	37	24.34	0.001***
No	458	157	34.28		16	3.49	
Occupation							
Employee	53	13	24.53	0.22	4	7.55	0.9
Housewives	557	185	33.21		49	8.80	
Marital status							
Single	105	39	37.14	0.3	4	3.81	0.040 *
Married	505	159	31.49		49	9.70	
History of abortion							
Yes	320	106	33.13	0.44	35	10.94	0.001 ***
No	290	92	31.72		18	6.21	
Restaurant food							
Yes	492	162	32.93	0.66	14	2.85	0.001***
No	118	36	30.51		39	33.05	

Females with contact with cats displayed a highly significant (P=0.001) seroprevalence of IgM antibodies compared to those without contact (37, 24.34% vs. 16, 3.49%). Conversely, females with contact with cats showed non-significantly higher IgG seroprevalence (41, 26.97% vs. 157, 34.28%) compared to those without such contact. Regarding occupation, non-significant differences (P=0.22 and P=0.09) were observed in the seroprevalence of both IgG (13, 24.53% vs. 185, 33.21%) and IgM (4, 7.55% vs. 49, 8.80%) antibodies among employees and housewives.

Married women exhibited a significant (P<0.04) difference in IgM seroprevalence (49, 8.80%) compared to unmarried individuals (4, 3.81%), while non-significant differences (P=0.3) were found in IgG seroprevalence between married and unmarried females (159, 31.47% vs. 39, 37.14%). Women with a history of abortion (35, 10.94%) and those consuming homemade foods (39, 33.05%) demonstrated highly significant (P=0.001) differences in IgM seroprevalence compared to individuals without a history of abortion (18, 6.21%) and those consuming restaurant food (14, 2.85%). Non-significant differences (P=0.44) were observed in IgG seroprevalence among individuals with a history of abortion (106, 33.13%) and those consuming homemade food (36, 30.51%) compared to those without a history of abortion (92, 31.72%) and those regularly consuming restaurant food (162, 32.93%).

## Discussion

The findings of this investigation demonstrate the endemicity of toxoplasmosis among the female population in Zakho city, Iraq, since high seroprevalence of both anti-*Toxoplasma* IgG (32.46%) and IgM (8.68%) was reported. The seroprevalence of both types of anti-*Toxoplasma *antibodies reported in the present study is higher than the seroprevalence rates of previous studies conducted in the same city. Mizuri and Mero reported seroprevalence rates of 11.58% and 0.63% for anti-*Toxoplasma *IgG and IgM, respectively [9]. Similarly, Abdulla and colleagues reported rates of 12.8% and 4.8% for IgG and IgM antibodies, respectively [10]. On the other hand, higher seroprevalence rates in females have been reported in previous studies from various parts of the country. In Erbil, rates of 37.5% and 34.18% for IgG and 9.13% and 10.86% for IgM, respectively, have been reported [21]. In Duhok city, Ramadhan and Sarkees have conducted a seroprevalence survey among female undergraduate students [22], and Salih and colleagues [23] conducted a study among healthy women and reported rates of 44.4% and 35.61% for IgG and 11.1% and 0.76% for IgM, respectively, while much higher rates for both IgG and IgM at 51.5% and 45%, respectively, have been reported in Kirkuk city [24].

The high seroprevalence rates of anti-*Toxoplasma *antibodies in the current study might be attributed to the characteristics of the enrolled participants. Since 15% of them were inhabitants of displacement camps in Cham Mishko, Zakho city, such habitat is poor in hygienic measures; in addition, we observed a large number of straw cats dwelling in the camp. Furthermore, other factors that might contribute to raising the seroprevalence rates of anti-*Toxoplasma *antibodies among the given population are dietary habits, personal hygiene practices, behavioral risks, environmental conditions, socioeconomic status, and suboptimal personal hygiene practices [25].

Middle-aged females (46-55) showed the highest rate of IgG antibodies as compared to younger and older ages. This finding is somewhat consistent with two previous studies in this city; both studies reported the highest seroprevalence of anti-*Toxoplasma *IgG and IgM antibodies among those aged 33-45 and 31-35 years, respectively [9,26]. Furthermore, studies from other countries also reported the highest rate of IgG seropositivity among middle ages: 25-32 years in Pakistan [27], 36-45 years in Duhok [23], 33-40 years in Nigeria [28], and 31-40 years in Iran [29].

The present study findings contradict a study performed in Erbil, which reported the highest seropositivity of IgM among young-aged females [30]. The highest seroprevalence rates reported in the present study among middle-aged females might be due to the possibility of specific lifestyle activities. Furthermore, the high significant increase in the seroprevalence of IgG with age is consistent with studies performed among pregnant women in the United States and Palestine [31,32]. The levels of IgG seropositivity are intricately linked to pathogen exposure. It's believable that younger individuals, having had fewer encounters with pathogens, exhibit lower baseline IgG levels. Conversely, in the elderly, reduced exposure to new pathogens may contribute to a decline in specific antibody production, including IgG [33]. Certainly, caution is necessary when explaining the increased IgG+ seroprevalence in middle age groups compared to both younger and older age groups. This interpretation should be approached with care due to the limitations imposed by a small number of participants within a restricted geographic area.

However, the seroprevalence of IgM in the present study was higher among young ages (18-25 years) compared with older ages, even though this difference was statistically non-significant. This finding is in agreement with other studies that also reported high seropositive IgM antibodies among younger ages. The lowest seroprevalence of anti-*Toxoplasma *IgM was observed among the age group 14-24 years which was 13.43% [21], 18-28 years which was 1% [34], 16-25 years which was 1.19 % [23], and 2-20 years which was 14.2 % [35]. However, in a study conducted in Erbil province, an IgM seropositive of young women sera was higher than older age women [30]. The lack of differences in the seroprevalence of IgM antibodies among different ages might be due to the chances of exposure to infection sources.

As regards residence, highly significant differences were reported in the seroprevalence of both types of antibodies between females from different residential areas with the highest among those living in displacement camps followed by rural inhabitants. This is consistent with studies on females in Duhok [36] and Erbil [30], who reported a notably higher seroprevalence of anti-*Toxoplasma *antibodies among females in rural residents compared with urban areas. On the other hand, the present study findings contradict a study in Iran that demonstrated a higher seroprevalence among females living in urban areas as compared to their rural counterparts [37]. While Hamad and Kadir, in Erbil, didn't find any significant differences between seropositivity of anti-*Toxoplasma *antibodies between females living in different residential areas [21], in the present study, several factors can contribute to a higher seroprevalence of anti-*Toxoplasma *antibodies between camp residents and other areas. The most probable factors are the high population densities, poor sanitation, limited healthcare facilities, poor health education, and abundance of stray cats. All these factors may contribute to contracting infectious diseases including toxoplasmosis. Kamal and colleagues stated that in Egypt community-based health programs have been implemented in many rural areas to address specific health concerns among the population [38].

Other factors, such as contact with cats, occupation, marital status, history of abortion, and consumption of restaurant foods, did not exhibit any association with IgG seropositivity among females in Zakho city. The lack of association between marital status and occupation agrees with the findings of previous studies, in Iraq [9] and Iran [39]. Similarly, being in contact with cats did not show any correlation with *T. gondii* IgG seropositivity among healthy blood donors in Iran [40,41]. Moreover, data from previous studies indicated a non-significant correlation between a "history of abortion" [42,43] or regularly consuming restaurant food [40,44] and chronic toxoplasmosis infection in women.

In contrast, regarding factors associated with *T. gondii* infection in the current study population, a positive association was observed between IgM seropositivity of *T. gondii *and contact with cats. The domestic cat serves as the final host for *T. gondii*, and the transmission may involve flies and cockroaches acting as mechanical vectors [45]. This finding is consistent with studies conducted in Iraq [9], as well as in Iran [46,47]; all these studies indicated the presence of significant associations between exposure to cats and the seroprevalence of anti-*Toxoplasma *IgM antibodies in the sera of tested women.

On the contrary, other studies didn't find any correlation between cat contact and the seroprevalence of anti-*Toxoplasma *IgM antibodies [26,48] in Duhok. This discrepancy might be attributed to the uncontrolled movement of cats or poor hygiene application; it is known that infected cats can excrete oocysts for up to two weeks during infection and individuals can become infected within five days. Additionally, the oocysts can survive for more than a year in the environment [49,50].

Furthermore, the rate of acute infections was slightly higher in married females compared to unmarried ones. This finding agrees with a study conducted in Cameroon, which demonstrated an association between marital status and IgM seropositivity [51]. On the other hand, Ramadhan and Sarkees, in Duhok, report higher seroprevalence of anti-*Toxoplasma *IgM among single undergraduate female students than married ones [22].

Meanwhile, Mizuri and Mero, in Zakho [9], and Ayeah and colleagues, in Cameron [52], didn't find any significant association between marital status and the seroprevalence of anti-*Toxoplasma *IgM antibodies among infected women. This discrepancy might be attributed to variations in the individuals who participated in the study and the geographical areas.

In the current study, occupation has a non-significant association with the seroprevalence of IgM antibodies. This is consistent with the findings conducted in Zakho [9], in Duhok [53], and in Erbil [54]; in all of these studies, non-significant differences were reported among various occupations. However, some studies reported higher seroprevalence of IgM among housewives compared with student and employed females, in Duhok [23] and in Erbil [30]. 

Moreover, the results of this study reveal a highly significant difference in IgM seropositivity between women with a history of abortions and those without. This finding is in line with studies that demonstrated the presence of high seroprevalence of anti-*Toxoplasma *IgM antibodies among women with a history of repeated abortions [26,30,43,55-57], while some studies didn't report any significant association between anti-*Toxoplasma *IgM antibodies and abortion [21,58]. Associating toxoplasmosis with recurrent abortions poses challenges as it would require a large cohort of women with toxoplasmosis to assess the incidence, otherwise unexplained recurrent abortions compared to controls.

Finally, in the present study, a positive association was observed between acute *T. gondii* infection and females who consumed restaurant meals. This finding agrees with [59], who reported a highly significant (P<0.001) association between acute infection and having lunch at the institution's restaurant. When considering the types of food served in restaurants or shops, there was a notable association between consuming raw salad [59], milk [60,61], and meat [62] and the occurrence of illness. It is likely that vegetables or meats served in restaurants can be considered as sources of infection.

The present study on the seroprevalence of *T. gondii* faced some limitations. The notable constraint was the insufficient representation of rural areas in the collected data, which may impact the generalizability of the current findings to broader populations. Additionally, employing a broader range of serological tests that can target other antigens could provide a more nuanced perspective on the prevalence of different strains, enhancing the overall robustness of the findings. Furthermore, a survey for gathering information on other diseases in participants could offer valuable insights into potential comorbidities and associations that might influence *T. gondii* seroprevalence. Despite these limitations, this study lays the groundwork for future research endeavors aimed at refining our understanding of *T. gondii* epidemiology.

## Conclusions

A high rate of seroprevalence of anti-*Toxoplasma gondii *IgG and IgM antibodies was recorded among females enrolled in this study. Significant associations were found between the acute infection of *Toxoplasma gondii *and some factors such as residence, contact with cats, a history of abortion, marital status, and consuming restaurant meals, but non-significant associations were found between age, occupational status, and acute infection. This aligns with the existing literature and emphasizes the importance of considering these variables in the context of toxoplasmosis transmission. On the other hand, the present study findings did not indicate the presence of any significant associations between chronic *Toxoplasma *infection and the abovementioned factors except age and residence, suggesting that the risk factors contributing to chronic infections may be universal and not influenced by sociodemographic characteristics. Therefore, factors such as the environment, genetics, or general health status might play a more prominent role.

## Appendices

**Table 4:** Patient's questionnaire.

Number:	Date of sampling: / /20	Location of sample collection:
Variables	Information	Notes
Gender	Female Male	
Age		
Residency	Urban Rural Camp	
Marital status	Single Married	
Occupation	Employee Housewife	
Presence and contact with cats	Yes No	
History of abortion	Yes No	
Eating restaurant food	Yes No	
